# Chemical Composition of Essential Oils of *Thymus* and *Mentha* Species and Their Antifungal Activities

**DOI:** 10.3390/molecules14010238

**Published:** 2009-01-07

**Authors:** Marina D. Soković, Jelena Vukojević, Petar D. Marin, Dejan D. Brkić, Vlatka Vajs, Leo J. L. D. van Griensven

**Affiliations:** 1 Institute for Biological Research "Siniša Stanković", Bulevar despota Stefana 142, 11000 Belgrade, Serbia; 2 Institute of Botany, Faculty of Biology, University of Belgrade, Takovska 42, 11000 Belgrade, Serbia; 3 Janssen-Cilag, Department of Johnson-Johnson S.E., Bulevar Mihajla Pupina 248, 11000 Belgrade, Serbia; 4 Faculty of Chemistry, University of Belgrade, Studentski trg 3, 11000 Belgrade, Serbia; 5 Plant Research International, Wageningen University and Research, P.O. Box 16, 6700 AA Wageningen, The Netherlands

**Keywords:** *Thymus vulgaris*, *T. tosevii*, *Mentha spicata*, *M. piperita*, Essential oils, Menthol, Thymol, Carvacrol, Carvone, Antifungal activity, Micromycetes

## Abstract

The potential antifungal effects of *Thymus vulgaris* L., *Thymus tosevii* L., *Mentha spicata* L., and *Mentha piperita* L. (Labiatae) essential oils and their components against 17 micromycetal food poisoning, plant, animal and human pathogens are presented. The essential oils were obtained by hydrodestillation of dried plant material. Their composition was determined by GC-MS. Identification of individual constituents was made by comparison with analytical standards, and by computer matching mass spectral data with those of the Wiley/NBS Library of Mass Spectra. MIC’s and MFC’s of the oils and their components were determined by dilution assays. Thymol (48.9%) and *p*-cymene (19.0%) were the main components of *T. vulgaris,* while carvacrol (12.8%), α-terpinyl acetate (12.3%), *cis*-myrtanol (11.2%) and thymol (10.4%) were dominant in *T. tosevii.* Both *Thymus* species showed very strong antifungal activities. In *M. piperita* oil menthol (37.4%), menthyl acetate (17.4%) and menthone (12.7%) were the main components, whereas those of *M. spicata* oil were carvone (69.5%) and menthone (21.9%). *Mentha* sp. showed strong antifungal activities, however lower than *Thymus* sp. The commercial fungicide, bifonazole, used as a control, had much lower antifungal activity than the oils and components investigated. It is concluded that **e**ssential oils of *Thymus* and *Mentha* species possess great antifungal potential and could be used as natural preservatives and fungicides.

## Introduction

Moulds have various health effects. Excessive mould growth in the human environment needs to be taken care of, regardless of the species, as it may lead to increased allergies, toxicity, and house/building structural problems. Some *Aspergillus* species are able to produce mycotoxins such as aflatoxins [1] that are potent hepatocarcinogens in animals and humans [2]. Therefore, the presence of toxigenic fungi in foods and grains presents a potential hazard to human and animal health. Superficial fungal infections, dermatomycoses, are probably the most common communicable fungal disease affecting humans. They have become a serious problem in immunocompromised patients [3]. Fungal infections remain a therapeutic problem despite the availability of a number of treatments. Being largely synthetic and non-biodegradable, these agents can cause adverse effects and may have residual toxicity. As the number of reported cases of food-associated infections continues to increase, food safety is a fundamental concern of both consumers and the food industry. As aromatic plants, herbs and spices have been used for ages both as flavouring agents and as preservatives of food, they may be effective sources of biodegradable fungitoxicants without harmful side effects. In the present study, a search was made to find essential oils that could safely be used as natural alternatives for chemical fungicides/fungistatics. 

Various *Mentha* and *Thymus* species have been credited with a long list of pharmacological properties. They have been used for their flavours in cooking, in folk medicine as antiseptic and as antimicrobial agents [4]. We chose these species for investigation of the antifungal activities of their oil. The essential oil of *T. vulgaris* is a known antiseptic, antiviral, antimicrobial agent [4,5,6]. The essential oil of *T. tosevii* contains a high amount of thymol and carvacrol and is also used in ethnobotany [7]. Its pharmacological activities are unknown. The present study reports the antifungal activities of the essential oils of two *Thymus* and two *Mentha* species and their main components against soil-borne pathogens, food storage fungi, mycotoxicogenic species, phytopathogens and opportunistic human pathogens. 

## Results and Discussion

### The composition of Thymus sp. essential oils and their antifungal effects

**Figure 1:**
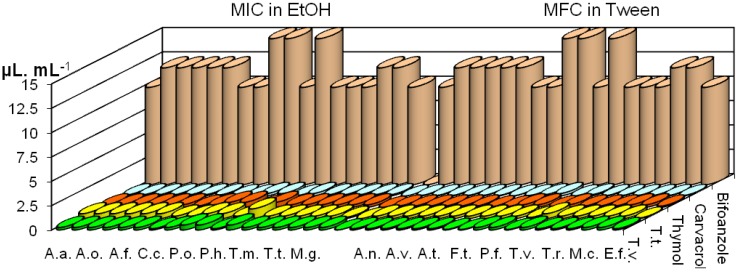
MIC (μL∙mL^-1^) in EtOH (macrodilution method) of oils of *T. vulgaris* and *T. tosevii* and their components and MFC (μL∙mL^-1^) in Tween^®^ (microdilution method).

We have identified 27 components, accounting for total 97.2% of the oil, in the essential oil of *T. vulgaris*. The main components were thymol (48.9%) and *p*-cymene (19.0%) (Table 1). The essential oil of *T. vulgaris*, tested by macrodilution method, showed very strong antifungal activity. A concentration of 0.25 μL/mL of oil inhibited *Alternaria alternata, Fusarium tricinctum,* all *Aspergillus* species and dermatomycetes. *Phomopsis helianthi* and *Cladosporium cladosporioides* were inhibited at lower concentrations (0.125 μL/mL). The essential oil dissolved in Tween^®^ showed better antifungal potential, with MIC 0.05 μL/mL. *Penicillium* species and *Trichoderma viride* were the most resistant fungi towards this oil; MIC in ethanol dissolved oil was 0.5 μL/mL, and 0.25 μL/mL when the oil was dissolved in Tween^®^. MICs of *T. vulgaris* oil in ethanol were 0.125-0.5 μL/mL, and 0.05-0.25 μL/mL in Tween^®^. Thymol showed the same antifungal potential as the oil, while carvacrol exhibited a slightly better effect, with MICs of 0.05-0.25 μL/mL in ethanol and 0.02-0.125 μL/mL in Tween^®^, (Figure 1). In agreement with earlier publications [8,9,10,11,12,13] the essential oil of *T. vulgaris* showed very strong antifungal activity at low concentrations, 0.05-1.0 μL/mL. Strong antifungal activity of thymol and carvacrol were also reported in the literature [4,5,6,7,8,9,10,11,12,13,14,15,16,17,18]. Previous investigations of Thyme oil by our group [19] showed very high activity against the three major pathogens of the button mushroom, *Agaricus bisporus,* i.e. the fungi *Verticillium fungicola* and *Trichoderma harzianum* and the bacterium *Pseudomonas tolaasii.* This oil also showed very strong antibacterial activity against food spoilage bacteria [20]. From the essential oil of *T. tosevii* we identified 42 components, 97.3% of the total amount. Carvacrol (12.8%), α-terpinyl acetate (12.3%), *cis*-myrtanol (11.2%) and thymol (10.4%) were the dominant components (Table 1). Earlier analysis has shown great variability in the presence of dominant components of *T. tosevii* oil; geraniol (0.15-32.66%), thymol (19.43-48.19%), geranyl acetate (0.04-11.60%), carvacrol (1.36-10.58%), linalool (0.25-20.92%), *p*-cymene (3.79-10.98%), depending on where the plants had been collected [7].

**Table 1:** Composition of the essential oils of *Mentha* and *Thymus* species.

Components	*Mentha spicata %*	*Mentha piperita %*	*Thymus tosevii %*	*Thymus vulgaris* %	*RI*
Tricyclene	0.3	-	-	-	926
α-Thujene	0.1	-	-	1.8	931
α-Pinene	0.1	-	-	1.2	939
Camphene	-	-	-	0.8	948
Sabinene	0.7	2.5	0.3	0.6	973
β-Pinene	0.4	-	0.2	0.4	980
β-Myrcene	2.3	0.5	-	1.1	991
3-Octanol	-	0.1	-	-	993
α-Terpinene	-	0.1	0.2	0.7	1018
*p*-Cymene	0.5	0.1	3.8	19	1026
Limonene	5.8	6.9	0.4	0.5	1030
1,8-Cineole	3	5.6	-	0.7	1031
*cis*-Ocimene	-	0.1	-	-	1040
*trans*-Ocimene	-	0.2	0.6	1.3	1050
γ-Terpinene	1.4	0.3	-	4.1	1068
*cis*-Linalool oxide	-	-	-	-	1072
Fenchone	-	-	-	-	1087
α-Terpinolene	0.3	0.1	0.2	-	1088
Linalool	-	0.2	2.3	0.7	1098
Camphor	-	-	0.2	0.2	1143
Menthone	21.9	12.7	-	-	1154
Menthofuran	-	6.8	-	-	1164
Borneol	-	-	2	1.7	1165
Menthol	0.5	37.4	-	-	1173
Terpin-4-ol	0.7	-	0.2	1.8	1177
α-Terpineol	-	-	5.9	-	1189
*cis*-Dihydrocarvone	0.3	-	-	-	1193
*trans*-Ddihydrocarvone	0.5	-	-	-	1200
Isodihydrocarveol	-	-	-	-	1215
*trans*-Carveol	0.2	-	-	-	1217
Thymol methyl ether	-	-	0.5	0.2	1235
Neral	-	-	-	-	1240
Carvone	49.5	-	-	-	1242
Pulegone	-	1.2	-	-	1243
Carvacrol methyl ether	-	-	0.7	1.7	1244
Piperitone	0.6	0.8	-	-	1252
Geraniol	-	-	-	-	1253
Geranial	-	-	0.4	-	1267
*trans*-Anethole	0.5	-	-	-	1283
*cis*-Myrtanol			11.2	-	
Bornyl acetate	-	-	1.2	-	1285
Thymol	-	-	10.4	48.9	1290
Menthyl acetate	-	17.4	-	-	1294
Carvacrol	-	-	12.8	3.5	1298
α-Terpinyl acetate	-	-	12.3	-	1350
Geranyl acetate	-	-	17.9	-	1383
β-Bourbonene	1.3	0.4	-	-	1384
β-Elemene	-	-	-	-	1391
*trans*-Myrtanol acetate	-	-	7.9	-	
β-Caryophyllene	0.7	0.3	2.9	3.5	1418
α-*trans*-Bergamotene	-	-	-	-	1436
α-Guaiene	-	-	-	-	1439
(*Z*)-β-Farnesene		0.7	-	-	1443
α-Guaieneε	-	-	0.2	0.3	1454
Germacrene D	0.3	0.5	0.6	0.3	1480
Bicyclogermacrene	-	1.3	-	-	1495
Germacrene A	0.5	0.5	1.3	-	1503
δ-Cadinene	-	0.8	0.1	-	1524
α-Cadinene	-	-	-	2.2	1538
Spatulenol	-	-	0.1	-	1578
Caryophyllene oxide	-	-	0.5	-	1581
Viridiflorol	-	0.2	-	-	1590
Total	92.4	97.7	97.3	97.2	

- not detected

The essential oil of *T. tosevii* possessed slightly lower antifungal potential than *T. vulgaris* oil. MICs and MFCs of this oil dissolved in ethanol were 0.25-1.0 μL/mL, and 0.125-0.5 μL/mL in Tween. *Phomopsis helianthi* was the most susceptible fungus, while *Trichoderma viride* was the most resistant species for this oil (Figure 1). There is no published data about the antifungal activity of *T. tosevii* oil. 

MICs of these oils were the same in macro- and microdilution methods, and the same as MFCs obtained by microdilution method, which means that concentrations that stopped fungal growth also killed the fungi. The commercial fungicide bifonazole, which was used as a control, possessed much higher MICs (8.0-15.0 μL/mL) than the essential oils and components investigated (Figure 1). The lower antifungal effect of *T. tosevii* oil in comparison with *T. vulgaris* oil can be explained by its lower amount of thymol and its precursors (p-cymene and γ-terpinene) and by the higher percentage of acetates (α-terpinyl acetate and geranyl acetate) which may lead to lower antifungal potential [18].

*A.a. - Alternaria alternata, A.n. - Aspergillus niger, A.o. - Aspergillus ochraceus, A.v. - Aspergillus versicolor, A.f. - Aspergillus flavus, A.t. - Aspergillus terreus, C.c. - Cladosporium cladosporioides, F.t. - Fusarium tricinctum, P.o. - Penicillium ochrochloron, P.f. - Penicillium funiculosum, P.h. - Phomopsis helianthi, T.v. - Trichoderma viride, T.m. - Trichosporon mentagrophytes, T.r. - Trichophyton rubrum, T.t. - Trichophyton tonsurans, M.c. - Microsporon canis, M.g. - Microsporon gypseum, E.f. - Epidermophyton floccosum.*

Comparing the previous data with the chemical composition of the oils, it becomes evident that there is a relationship between the high activity of the *Thymus* type oils and the presence of phenol components, such as thymol and carvacrol. The high antifungal activity of these essential oils could be explained by the high percentage of phenol components. It seems possible that phenol components may interfere with cell wall enzymes like chitin synthase/chitinase as well as with the α- and β- glucanases of the fungus [21]. Consequently, the high content of phenol components may account for the high antifungal activity of oils [16]. From our results it can be seen that essential oils of *Thymus* species and carvacrol and thymol have very high antifungal activities, even higher than the commercial fungicide bifonazole.

### The composition of Mentha sp. essential oils and their antifungal effects

**Figure 2:**
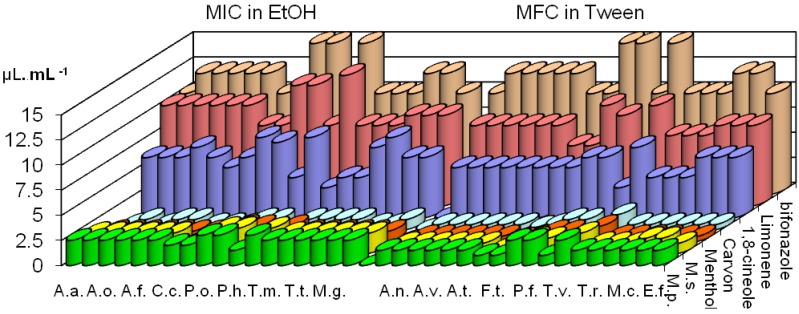
MIC (μL∙mL^-1^) in EtOH (macrodilution method) of oils of *M. piperita* and *M. spicata* and their components and MFC (µL∙mL^-1^ ) in Tween^®^ (microdilution method).

In *M. piperita* essential oil 26 components were detected and identified (97.7%). Menthol (37.4%), menthyl acetate (17.4%) and menthone (12.7%) were the main components in this oil (Table 1). In essential oil of *M. spicata* 27 components were identified (92.4%). The main components were carvone (49.5%) and menthone (21.9%) (Table 1). The results of antifungal activities of both essential oils and components are presented in Figure 2. The essential oil of *M. spicata* showed the same fungistatic activity in macro- and microdilution method with MICs of 1.0-2.5 μL/mL in ethanol and 0.5-1.5 μL/mL in Tween^®^. MICs of *M. piperita* essential oil were higher, 1.5-3.0 μL/mL in ethanol and 1.0-2.5 μL/mL in Tween^®^. Figure 2 shows that that essential oil of *M. spicata* possesses greater fungistatic activity than *M. piperita* oil. Minimum fungicidal concentrations (MFC) of *M. spicata* essential oil obtained by microdilution method, diluted in ethanol and Tween^®^ were 1.0-2.5 μL/mL and 0.5-2.5 μL/mL, respectively. These values (MFC) for *M. piperita* essential oil were 1.5-3.0 μL/mL and 1.0-2.5 μL/mL. It can be seen that MFCs of essential oil of *M. spicata* are higher than MFCs of essential oil of *M. piperita* (Figure 2). 1,8-cineole diluted in ethanol inhibited fungal growth in concentrations of 3.0-8.0 μL/mL, while in Tween^®^, MICs were 2.0-7.0 μL/mL. 

A.a. - Alternaria alternata, A.n. - Aspergillus niger, A.o. - Aspergillus ochraceus, A.v. - Aspergillus versicolor, A.f. - Aspergillus flavus, A.t. - Aspergillus terreus, C.c. - Cladosporium cladosporioides, F.t. - Fusarium tricinctum, P.o. - Penicillium ochrochloron, P.f. - Penicillium funiculosum, P.h. - Phomopsis helianthi, T.v. - Trichoderma viride, T.m. - Trichosporon mentagrophytes, T.r. - Trichophyton rubrum, T.t. - Trichophyton tonsurans, M.c. - Microsporon canis, M.g. - Microsporon gypseum, E.f. - Epidermophyton floccosum

Menthol showed the same fungistatic and fungicidal activities in both methods, with MICs of 0.25-1.5 μL/mL in ethanol and 0.05-1.0 μL/mL in Tween^®^ (Figure 2). Limonene possessed moderate fungistatic activity with MICs 8.0-12.0 μL/mL in ethanol and 5.0-9.0 μL/mL in Tween^®^. MICs of limonene obtained in microdilution method were 6.0-11.0 μL/mL in ethanol and 5.0-9.0 μL/mL in Tween^®^. MFCs of this component were 7.0-11.0 μL/mL in ethanol and 5.0-10.0 μL/mL in Tween^®^. Carvone showed higher antifungal activities than previous components. MICs and MFCs of this component were 0.25-1.0 μL/mL in ethanol and 0.05-0.5 μL/mL in Tween^®^. There is no effect of ethanol and Tween against tested micromycetes. The commercial fungicide (bifonazole), which was used as a control showed lower antifungal potential than the essential oils investigated (Figure 2). 

The differences between antifungal activities of these two essential oils could be due to different chemical composition of essential oils. The greater antifungal potential of *M. spicata* essential oil could be explained by the presence of carvone, which possesses very strong antifungal activity [14,16]. The essential oil of *M. piperita* possesses menthol and 1,8-cineole as main components, which also exhibited very good antifungal properties but lower than carvone [18]. Carvone has better antifungal properties because of its high water solubility. One of the reasons for lower antifungal activity of *M. piperita* essential oil could be the large amount of menthyl acetate, which causes a decrease of antifungal properties [18]. 

Earlier investigations of the antimicrobial activity of *Mentha spicata* and *M. piperita* oils showed strong activity against the three major pathogens of the button mushroom, *Agaricus bisporus,* i.e. the fungi *Verticillium fungicola* and *Trichoderma harzianum* and the bacterium *Pseudomonas tolaasii* [19]. Both *Mentha* oils tested inhere showed strong antibacterial activity against a variety of bacteria [20]. Our results are in accordance with previous investigations of antifungal activities of *Mentha* essential oils. Previous results showed a fungicidal effect of *M. spicata* essential oil against human pathogens in concentration of 0.25 -2.0 μL/mL, while this oil possessed lower antifungal potential against phytopathogenic species [9,16,22]. The results obtained by both methods suggested that carvone and menthol possessed greater antifungal activities than other compounds investigated, while limonene showed the lowest antifungal activity. Previous results of investigation of antifungal properties had shown that hydrocarbon monoterpenes had the lowest antifungal activity, larger antifungal potential could be due to the presence of oxygenated terpenes or of those with phenolic structures [9,14,16,29]. The hydrocarbons tend to be relatively inactive regardless of their structural type, and this inactivity is closely related to their limited hydrogen binding capacity and water solubility [17]. Ketones, aldehydes and alcohols showed activity but with differing specificity and levels of activity, which is in connection with the functional groups present but also associated with hydrogen-binding parameters in all cases.

From our results it can be seen that MICs and MFCs are generally lower for all the essential oils and components investigated in microdilution method. The low water solubility of the oil and its components limit their diffusion through the agar medium. Only the more water-soluble components, such as carvone or 1,8-cineole diffuse into the agar. The hydrocarbon components either remain on the surface of the medium or evaporate [18]. That could be the reason for better results obtained by the microdilution method. Broth method, carried out in microtitre trays, has the advantage of lower workloads for a larger number of replicates and the use of small volumes of the test substance and growth medium. In this method dilution of the oil is better and there is no agar in the medium, which both enable better diffusion through the liquid medium.

### Solvents affect the antifungal effects of Thymus and Mentha species essential oils

Also, it can be seen that the essential oils and components investigated showed greater antifungal activities when they are diluted in Tween^®^. Non-ionic emulsifiers, such as Tween^®^ 20 or 80, are relatively inactive when tested alone and have been widely reported in the literature for their use as emulsifying agents [18]. It was achieved that dispersion of the oil and components is better than when ethanol was used as a solvent.

However, essential oils and all the components investigated showed inhibitory effects against all 17 fungi tested. The mycelial growth of test fungi responded differently to the investigated essential oil, which indicated that oils may have different modes of action or that the metabolism of some fungi was able to better overcome the effect of the oil or adapt to it. Phytopathogen *Phomopsis helianthi* was the most susceptible to the investigated essential oils, while *Trichoderma viride* and *Penicillium* species were the most resistant species (Figure 1 and Figure 2).

### Thymus and Mentha species essential oils can be used safely

In an earlier study of one of us [28], it was found that the LC50 value of orally applied Thyme oil in rat was 9543.5 μL kg^-1^ bodyweight. The LD_50_ values of pyrethrin (350-500 mg kg^-1^) and carvone (1640 mg kg^-1^) are well below that of Thyme oil [29]. *Mentha piperita* and *Thymus vulgaris* as well as their oils and the major oil components are generally regarded as safe and full toxicology has been obtained [30]. 

Taken together, these results suggest that the essential oils investigated here could find practical application in the prevention and protection of fungal infections of plants, animals and humans. Essential oils could safely be used as preservative materials on foods for protection to fungal infection, since they are natural, and mostly non-toxic to humans. The selected plant species are popular culinary herbs, and their essential oils have been used extensively for many years in food products, perfumery, and dental and oral products. 

## Conclusions

The essential oils of *T. vulgaris*, *T. tosevii* and of *M. piperita* and *M. spicata* are highly active as fungitoxicants and could safely be used as natural preservatives to replace synthetic fungicides in the prevention and cure of some plant, human and animal fungal disease.

## Experimental

### Materials

Essential oil of *Thymus vulgaris* in commercial samples was obtained from the Institute for Medicinal Plant Research “dr Josif Pančić“, Belgrade. *T. tosevii* was collected from the South of Serbia (Vranje) in 2007. The voucher specimen (1211963) was deposited at the Herbarium of Botanical garden “Jevremovac”, Faculty of Biology, University of Belgrade. Essential oils were isolated from air-dried aerial part by hydrodistillation in a Clevenger type apparatus for two hours.

The fungi used in this study were: *Aspergillus niger* (ATCC 6275),* A. ochraceus* (ATCC 12066),* A. versicolor* (ATCC 11730),* A. flavus* (ATCC 9170),* A. terreus* (ATCC 16792),* Alternaria alternata* (ATCC 13963),* Penicillium ochrochloron* (ATCC 9112),* P. funiculosum* (ATCC 10509),* Cladosporium cladosporioides* (ATCC 13276),* Trichoderma viride* (IAM 5061),* Fusarium tricinctum* (CBS 514478),* Phomopsis helianthi* (ATCC 201540), and five dermatomycetes,* Microsporum canis, Epidermophyton floccosum, Trichophyton rubrum, T. mentagrophytes* and* T. tonsurans*. The moulds were from Mycoteca of the Mycological Laboratory, Department of Plant Physiology, Institute for Biological Research, Belgrade. Dermatomycetes were isolated directly from the patients at the Center for preventive Medicine, MMA, Belgrade. The fungi were maintained on potato dextrose agar (PDA), malt agar (MA) and Sabouraud agar (SBA), [26]. The cultures were stored at +4°C and subcultured once a month.

### Instrumentation

The essential oils were investigated for their composition by the use of analytical GC/FID and GC/MS techniques. For this purpose a HP 5890 series II gas chromatograph, equipped with split-splitless injector, fused silica capillary column (25 m x 0.32 mm), coated with cross-linked methyl silicone gum (0.5 μm film thickness), and FID was employed. Essential oil solutions in ethanol (1%) were injected in split mode (1:30). Injector was heated at 250^o^C, FID at 300 °C, while column temperature was linearly programmed from 40-280 °C (4 °C /min). GC/MS analyses were carried out on a HP-GCD, equipped with split-splitless injector, fused silica capillary column (50 m x 0.2 mm) PONA, coated with cross-linked methyl silicone gum (0.5 μm film thickness). The chromatographic conditions were as above. Transfer line (MSD) was heated at 280 °C. EIMS spectra (70eV) were acquired in scan mode in m/e range 40-300. Identification of individual constituents was made by comparison of their retention times with those of analytical standards, and by computer searching, matching mass spectral data with those held in Wiley/NBS Library of Mass Spectra. For quantification purposes area percent reports obtained by FID were used.

### Tests for antifungal activity

*Macrodilution method:* In order to investigate antifungal activity of essential oils and their components, the modified mycelial growth test with malt agar was used [27]. The minimum inhibitory concentration (MIC) of investigated components necessary for the complete inhibition of mycelial growth of the fungal strain was determined. Different concentrations of essential oils and components were diluted in Petri dishes with malt agar (MA). All fungal species were tested in triplicate. Petri dishes with ethanol and bifonazole were used as a control. Essential oil was added into molten malt agar (MA) and poured into Petri dishes. The tested fungi were inoculated at the center of the plates. Plates were incubated for three weeks at room temperature, after this period MIC was determined.

*Microdilution method:* The modified microdilution technique was used [26,27]. The fungal spores were washed from the surface of agar plates with sterile 0.85% saline containing 0.1% Tween^®^ 80 (vol/vol). The spore suspension was adjusted with sterile saline to a concentration of approximately 1.0 x 10^5^ in a final volume of 100 μL per well. The inocula were stored at +4 °C for further use. Dilutions of the inocula were cultured on solid MA to verify the absence of contamination and to check the validity of the inoculum. Minimum inhibitory concentrations (MICs) determination was performed by a serial dilution technique using 96-well microtitre plates. Essential oils and compounds investigated were dissolved in malt medium broth with fungal inoculum. The microplates were incubated for 72 h at 28 °C. The lowest concentrations without visible growth (at the binocular microscope) were defined as concentrations, which completely inhibited fungal growth (MICs). The minimum fungicidal concentrations (MFCs) was determined by serial subcultivation of 2 μL into microtitre plates containing 100 μL of broth per well and further incubation for 72 h at 28 °C. The lowest concentration with no visible growth was defined as the MFC, indicating = 99.5 % killing of the original inoculum.
